# Buprenorphine misinformation and willingness to treat patients with opioid use disorder among primary care-aligned health care professionals

**DOI:** 10.1186/s13722-024-00436-y

**Published:** 2024-01-19

**Authors:** Berkeley Franz, Lindsay Y. Dhanani, O. Trent Hall, Daniel L. Brook, Cheyenne Fenstemaker, Janet E. Simon, William C. Miller

**Affiliations:** 1 Ohio University Heritage College of Osteopathic Medicine, Department of Social Medicine, Ohio University Athens, Heritage Hall 1, Athens, OH 45701-2979 USA; 2 Appalachian Institute to Advance Health Equity Science, Athens, OH USA; 3 Rutgers University School of Management and Labor Relations, Piscataway, NJ USA; 4 Department of Psychiatry and Behavioral Health, Ohio State University Wexner Medical Center, Columbus, OH USA; 5 Ohio State University College of Public Health, Columbus, OH USA; 6 Ohio University College of Health Sciences and Professions, Athens, OH USA; 7 Gillings School of Public Health , University of North Carolina-Chapel Hill, Chapel Hill, NC, USA

**Keywords:** Opioid-related disorders, Primary care, Misinformation, Buprenorphine, Addiction medicine

## Abstract

**Background:**

Buprenorphine is a highly effective medication for opioid use disorder that is underused by health care professionals (HCPs). Medications for opioid use disorder (MOUD) misinformation may be an important barrier to buprenorphine access, but most implementation strategies have aimed to reduce negative attitudes towards patients with opioid use disorder (OUD) rather than misinformation specific to buprenorphine use. In this study, we assessed the degree to which HCPs endorsed misinformation related to buprenorphine, and whether this is associated with willingness to provide care to patients with OUD.

**Methods:**

In September-December of 2022, we surveyed HCPs practicing in Ohio (n = 409). Our primary outcomes included a previously validated 5-item measure of HCP willingness to treat patients with OUD, and three other measures of willingness. Our key independent variable was a study-developed 5-item measure of endorsement of misinformation related to buprenorphine, which assessed beliefs in buprenorphine’s efficacy in managing withdrawal symptoms and reducing overdose deaths as well as beliefs about the role of buprenorphine in achieving remission. We computed descriptive and bivariable statistics and fit regression models predicting each outcome of interest.

**Results:**

On average, HCPs scored 2.34 out of 5.00 (SD = 0.80) on the composite measure of buprenorphine misinformation. 48.41% of participants endorsed at least one piece of misinformation. The most endorsed items were that buprenorphine is ineffective at reducing overdose deaths (M = 2.75, SD =0 .98), and that its use substitutes one drug for another (M = 2.41, SD = 1.25). HCP endorsement of buprenorphine misinformation significantly and negatively predicted willingness to work with patients with OUD (b = − 0.34; 95% CI − 0.46, − 0.21); intentions to increase time spent with this patient population (b = − 0.36; 95% CI − 5.86, − 1.28); receipt of an X-waiver (OR = 0.54, 95% CI 0.38, 0.77); and intention to get an X-waiver (OR: 0.56; 95% CI: 0.33−0.94).

**Conclusions:**

Misinformation is common among HCPs and associated with lower willingness to treat patients with OUD. Implementation strategies to increase MOUD use among HCPs should specifically counter misinformation related to buprenorphine.

*Clinical Trial Registration*: Clinicaltrials.gov, NCT05505227. Registered 17 August 2022, https://clinicaltrials.gov/ct2/show/NCT05505227

## Introduction

Buprenorphine, one of the three available evidence-based medications for opioid use disorder (MOUD) [1] is considered the gold standard in achieving remission and also shows remarkable efficacy in reducing a person’s risk of overdose [2]; death [3]; and infectious disease transmission [4–6]. Buprenorphine has similar efficacy to methadone, but can be prescribed in outpatient settings, significantly removing barriers to treatment [7]. Despite compelling evidence and proven benefits, the United States lacks enough buprenorphine prescribers, and the medication remains substantially underused [8]. Fewer than 6% of physicians and 3% of nurse practitioners and PAs received the previously-required X-waiver to prescribe this medication [9, 10]. A sobering result is that fewer than one in five patients with OUD currently receives MOUD [11].

Numerous barriers to buprenorphine prescribing have been documented at the individual- and policy-level that inhibit prescribing. Among the most examined have been restrictive regulation such as the recently removed DATA 2000 (X) waiver [12], as well as stigma and limited knowledge among clinicians [13]. Indeed, clinicians’ self-rated knowledge, comfort level, and ability to diagnose substance use disorders (SUDs) remain low and many physicians also perceive their medical training on substance use as insufficient [14, 15]. PCPs, in particular, report inadequate training in prescribing opioids, feel unprepared to treat OUD effectively, and find it stressful to manage patients with chronic pain [16]. Widespread stigma is also well-documented among health care professionals (HCPs) that interact with patients with OUD, including those who specialize in substance use treatment [18–22]. Such attitudes among HCPs may adversely affect health outcomes by reducing patient engagement in treatment [18, 23] and reducing willingness to prescribe MOUD [18, 22].

Understanding the full range of barriers is critical to developing implementation strategies to increase buprenorphine prescribing [25], but the current literature is limited in that it has not fully investigated the role of misinformation, or false or inaccurate information [26], as a barrier to prescribing buprenorphine. Importantly, misinformation is distinct from previous considerations of knowledge about buprenorphine in that knowledge considers the degree to which clinicians are uninformed about the mechanism of buprenorphine whereas misinformation captures their misbeliefs about its effectiveness and/or appropriateness for managing OUD. Stated differently, a lack of knowledge is an uncertainty about how or when to use buprenorphine, whereas misinformation reflects certainty in incorrect information about buprenorphine’s efficacy or value in treating OUD.

We argue that the absence of literature on the role of misinformation is of primary concern because misinformation about buprenorphine is widespread [27] and the endorsement of such beliefs may cause HCPs to falsely conclude that buprenorphine is not an effective or appropriate treatment for OUD. Common misinformation related to MOUD includes concerns that patients on MOUD are simply substituting one drug for another, or that their prescription enables patients’ addiction and does not truly constitute or facilitate recovery [28, 29]. Other misinformation may include beliefs that call into question the efficacy of buprenorphine at managing withdrawal symptoms or reducing the likelihood of overdose [30]. This misinformation has spread within communities of clinicians [7, 24, 31, 32] and the broader public [30, 33], and may undermine HCP willingness to provide evidence-based MOUD treatment. That is, when clinicians believe these misleading statements about buprenorphine, they may view it as a harmful treatment path instead of accurately identifying its clinical benefit for patients with OUD. For example, an endorsement of the common myth that buprenorphine substitutes one drug for another may lead clinicians to conclude that they are prescribing a medication that will only prolong a substance use disorder rather than help treat it.

The current study therefore aims to investigate the degree to which HCPs endorse misinformation related to buprenorphine and the relationship between that endorsement and critical treatment decisions. Since the significance of MOUD misinformation is not well understood, the question remains whether misinformation is a previously unexamined barrier to buprenorphine access. If true, the clinical importance of misinformation cannot be understated, as current implementation strategies will need to be revised to address not only HCP knowledge and stigma, but also MOUD misinformation. To address this question, we assessed buprenorphine misinformation among a large sample of HCPs and its association with willingness to treat patients with OUD, devote time to OUD treatment, and receive previously required training to be able to prescribe MOUD.

## Methods

### Study population

Our sample included 409 HCPs licensed to practice in the State of Ohio. We recruited HCPs to understand the extent to which OUD is currently managed in the primary care setting. Inclusion criteria included being an active HCP eligible to prescribe MOUD: physician, nurse practitioner, or physician’s assistant. The second criterion was practicing in primary care or an aligned specialty which has a high likelihood of coming into contact with patients with OUD; a medical practice that has an important role in OUD care coordination, such as family medicine, internal medicine, addiction medicine, obstetrics/gynecology, infectious disease, emergency medicine, and psychiatry [34]. We set an a priori goal of 400 participants based on power analyses. To increase participation and improve diversity in our sample, we recruited for the survey using multiple approaches: [1] we emailed 20,143 HCPs in the included disciplines using the State Board of Medical Licensing roster; [2] we advertised the study through several professional associations in Ohio; and [3] we worked with health professions training programs in the state to advertise the survey to alumni. Because we utilized state licensure rosters with many out-of-date email addresses (e.g. email addresses associated with the university where individuals trained), and because we relied on newsletters and listservs associated with professional organizations and health professions schools, calculating a response rate is not possible. We sent direct email invitations in batches, beginning in September 2022, and two reminder emails approximately 3 and 7 days after the original email invitation. The survey was closed when 400 participants had responded, on 12/23/22. All participants who completed the survey were eligible to receive a $20 Amazon gift card as compensation for their time. To maintain anonymity, participants were re-rerouted to a second survey, and asked to insert their email address and a random number generated in the first survey. Our study was approved by the [name redacted] internal review board, and all respondents provided electronic informed consent prior to participation.

### Data and measures

Our survey contained primarily closed-ended questions measuring attitudes and behaviors related to treating OUD in the primary care setting. We had four primary outcome measures, including willingness to treat patients with OUD, which was measured using a previously validated 5-item measure [35] with 5 coded as most willing (α = 0.90). Our second outcome measured whether participants had an active DEA X-waiver, using a binary Yes/No variable. Among those that did not, our third outcome measured interest in receiving a DEA X-waiver, also as a binary Yes/No variable. Our final outcome measure was the desire to increase the amount of time spent with patients with OUD. We created this variable by subtracting the current percentage of a participant’s time spent with patients with OUD from the preferred amount of time spent with this patient population.

Our focal independent variable was the Endorsement of Buprenorphine Misinformation Scale (EBMS). The EBMS is a 5-item measure containing common misinformation about buprenorphine, measured on a 5-point Likert scale ranging from extremely disagree to extremely agree, where higher scores indicate greater endorsement of misinformation. Scores of 4 and 5 on the EBMS were combined to indicate endorsement of misinformation for descriptive statistics. The EBMS was created for the current study based on common misconceptions about buprenorphine [30]. We computed an estimate of internal consistency reliability, and results demonstrated that the scale had adequate reliability (α = 0.77). An exploratory factor analysis suggested that all 5 items loaded onto a single factor with an eigenvalue of 2.64 (Table 1), which accounted for 53% of the variance. The lowest factor loading was 0.45.

**Table 1:** Factor loadings for the endorsement of buprenorphine misinformation scale

Item	Factor loadings
Buprenorphine is not effective at managing withdrawal symptoms	0.45
Buprenorphine is not effective at reducing overdose deaths	0.58
Buprenorphine simply substitutes one drug for another	0.84
Buprenorphine discourages patients from seeking long-term remission	0.85
Buprenorphine encourages patients to keep using opioids	0.81
Eigenvalue	2.64

Factor loadings were calculated by conducting a principal components analysis

Additional covariates included a measure of explicit bias towards patients with OUD, using a previously validated 8-item measure [36]. Responses are coded on a 5-point scale ranging from “Strongly Agree” to “Strongly Disagree,” with 5 coded as higher bias (α = 0.85). We also measured perceived training needs using a single item measure: “I feel that I have received adequate training for treating patients with opioid use disorder,” which had five response options ranging from “Strongly agree” to “Strongly disagree.” We measured HCP demographics, including gender, age, race, training credential, the average number of hours they work per week, and the county in which they work. Using data from the Ohio Department of Health, we constructed a measure of rurality using the county of practice (1 = Urban county, 2 = Partially rural county, 3 = Rural county) [37].

### Analysis

To assess the relationship between buprenorphine misinformation and willingness to work with patients with OUD, we first employed descriptive statistics to describe the sample, and then calculated Pearson correlation coefficients to estimate the bivariate relationships among our study variables. Next, we performed multivariable linear regression and logistic regression to estimate the unique relationship between buprenorphine misinformation and our outcome variables after accounting for other factors associated with willingness, including explicit bias and receipt of adequate training (e.g. age, training etc.). This offers a more robust investigation of the relationship between buprenorphine misinformation and the outcome of interest because it allows us to identify the unique contribution of the relationship while taking into consideration other variables that influence the outcome of interest. All statistical analyses were conducted using Stata 15.

## Results

### Descriptive statistics

Among the 409 HCPs who completed the survey, 29% (n = 121) worked as advanced practice registered nurses; 43% (n = 174) worked as physicians; and 27% (n = 109) worked as physician’s assistants. Approximately 60% (n = 245) were female, and the average age of respondents was 42.3 years old (SD = 11.96). Participants had been in practice for an average of 17.77 years (SD = 11.58) and worked an average of 41.3 h per week (SD = 13.14); Table 2). Nearly 62% (n = 265) of the sample practiced in an urban area, with 15.4% (n = 66) and 22.7% (n = 97) of participants practicing in partially rural and rural counties, respectively.

**Table 2:** Demographics of study participants (N = 404)

Variable	N	%
Gender		
Male	154	38
Female	245	61
Non-binary	5	1
Provider type		
Physician assistant	109	27
Nurse practitioner	121	29
Physician	174	43
Race		
White alone	330	82
Black or African American	12	3
Asian or Asian American	25	6
More than one race	15	4
Race not reported	22	5

On average, PCPs scored 2.34 out of 5.00 on the composite measure of buprenorphine misinformation (SD = 0.80). 48.41% of PCPs endorsed at least one piece of buprenorphine misinformation. The most endorsed misinformation about buprenorphine was that it substitutes one drug for another (M = 2.41, SD = 1.25); 25.67% of participants either somewhat or strongly agreed with this statement. The second most common misinformation was that buprenorphine is not effective at preventing overdose (M = 2.75, SD = 0.98), which was endorsed by 20.78% (n = 85) of participants. Nearly half of participants either somewhat or strongly agreed that they had received adequate training in the management of OUD. The average score for negative bias towards patients with OUD was 2.04 out of 5.00 (SD = 0.70).

### Bivariate

**Fig. 1:**
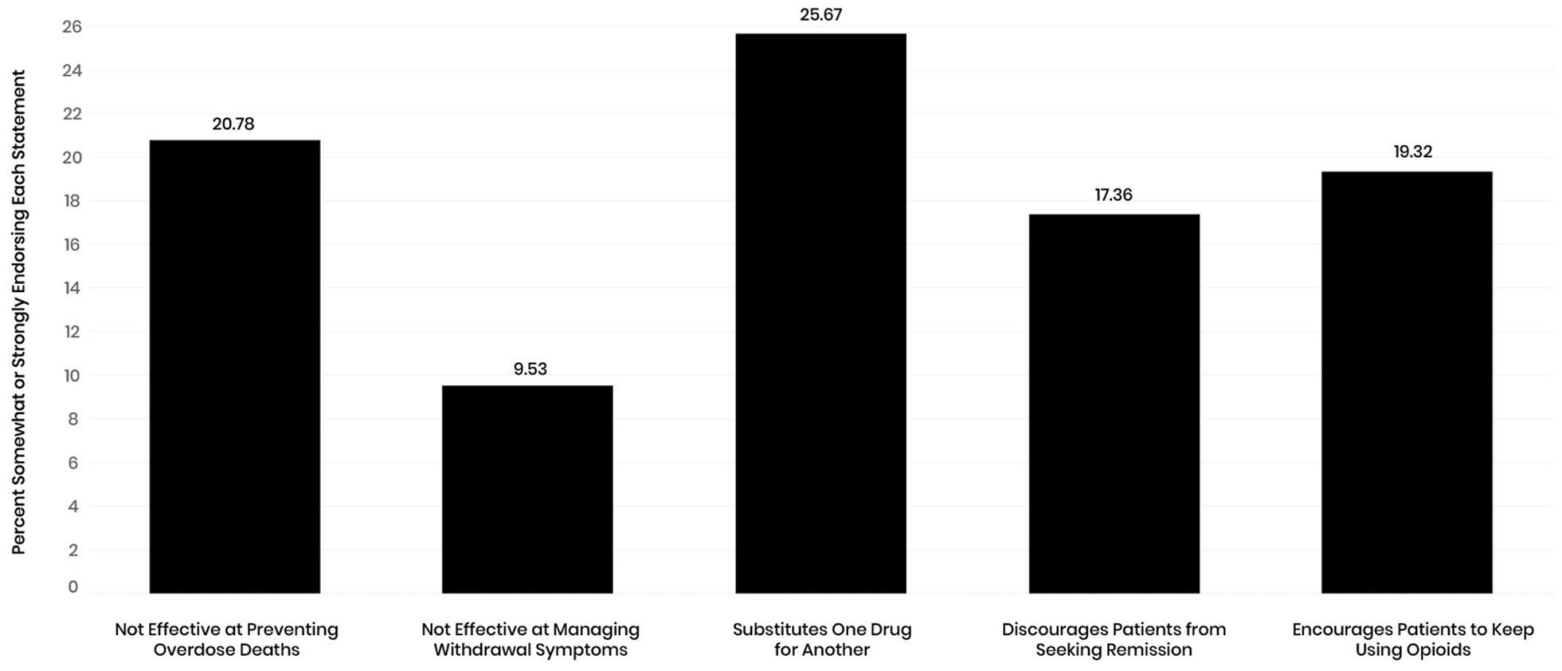
Support for different forms of buprenorphine misinformation

We first calculated Pearson correlation coefficients to examine the bivariate relationships between all study variables. Results indicated that endorsing buprenorphine misinformation was associated with a significant decrease in willingness to treat patients with OUD (r = − 0.44, p < 0.001), and a decrease in the preferred percentage of patients they manage who have OUD (*r* = − 0.20, p < 0.001) (Table 3). Participants who endorsed misinformation were significantly less likely to hold X waivers (r = − 0.29, p < 0.001) and to be interested in obtaining an X waiver to prescribe buprenorphine (r = − 0.19, p < 0.01) (Fig. 1).

**Table 3:** Bivariate correlations among study variables

Variable	M	SD	1	2	3	4	5	6	7	8	9	10	11	12	13	14	15
Gender	1.61	0.49															
Age	42.43	11.96	− 0.30*														
White alone	0.82	0.39	0.12*	0.03													
Nurse practitioner	0.30	0.46	0.33*	− 0.06	0.16*												
Physician	0.43	0.50	− 0.39*	0.31*	− 0.27*	− 0.57*											
Physician assistant	0.27	0.44	0.09	− 0.28*	0.14*	− 0.40*	− 0.53*										
Work hours	41.31	13.14	− 0.15*	0.06	− 0.06	− 0.19*	0.22*	− 0.05									
Years in job	18.61	12.35	− 0.06	0.05	− 0.07	0.00	0.11*	− 0.12*	0.03								
Rural location	1.61	0.83	0.00	0.11*	0.03	0.07	− 0.05	− 0.01	− 0.01	0.02							
Received training	3.17	1.35	− 0.14*	0.10	− 0.08	− 0.10*	0.06	0.04	0.05	0.02	0.06						
Bias	2.34	0.62	− 0.15*	0.06	− 0.13*	0.00	0.03	− 0.04	− 0.10	0.09	0.06	− 0.22*	(0.85)				
Buprenorphine misinformation	3.34	0.80	0.03	− 0.07	− 0.11*	− 0.10*	− 0.09	− 0.09	− 0.06	0.03	0.11*	− 0.26*	0.50*	(0.77)			
Willingness to treat OUD	3.13	0.86	0.03	− 0.08	− 0.01	0.07	− 0.09	0.03	− 0.01	− 0.07	0.05	0.31*	0.05	− 0.44*	(0.90)		
Interest in increasing time for OUD treatment	3.17	1.35	0.06	0.03	0.05	0.16*	− 0.13*	− 0.03	0.01	0.07	− 0.00	0.06	− 0.19*	− 0.20*	0.37*		
Receipt of X-waiver	0.48	0.50	− 0.09	0.11*	0.02	− 0.03	− 0.06	0.06	− 0.01	− 0.02	0.01	0.45*	− 0.29*	− 0.34*	0.38*	0.20*	
Interest in X-waiver	0.36	0.48	− 0.13	0.11	0.04	− 0.00	− 0.05	0.07	− 0.03	− 0.28*	0.01	− 0.03	− 0.14*	− 0.20*	0.46*	0.26*	

N = 404 with the exception of Interest in X-waiver (n = 269); alphas appear on the diagonal; gender is coded 1 = male and 2 = female; Rural location is coded 1 = urban, 2 = partially rural, 3 = rural, Receipt of and interest in X-waiver are coded 1 = yes and = no, Bias, buprenorphine misinformation, and willingness to treat OUD are measured on a 5-point scale, *p < 0.05

### Multivariable regression

After accounting for participant demographics, training, and bias, endorsing buprenorphine misinformation remained significantly and negatively associated with willingness to work with patients with OUD (β = − 0.234, 95% CI − 0.47, − 0.21). Endorsing more buprenorphine misinformation was also associated with less interest in increasing the percentage of patients managed with OUD (β = − 3.57, 95% CI − 5.86, − 1.29); and lower odds of having received an X-waiver (OR: 0.55, 95% CI 0.39, 0.78) (Table 4). Among those not currently holding X-waivers, endorsing buprenorphine misinformation was also significantly and negatively associated with interest in obtaining an X-waiver (OR = 0.56, 95% CI 0.34, 0.94). Having received adequate training on OUD was significantly associated with greater willingness to work with patients with OUD (β = 0.15, 95% CI 0.09, 0.22), and higher odds of having received an X-waiver (OR = 1.93, 95% CI 1.60, 2.33). Negative bias towards patients with OUD was significantly and negatively associated with willingness to work with this patient population (β = − 0.53, 95% CI − 0.63, − 0.33), as well as lower odds of being interested in receiving the X-waiver (OR = 0.56, 95% CI 0.40, 0.88).

**Table 4:** Regression Results

	Interest in X-waiver (n = 202)			Received X-waiver (n = 402)			Interest in increasing time for OUD treatment (*n *= 402)			Willingness to treat OUD (n = 402)		
	OR	SE	95% CI	OR	SE	95% CI	Coef.	SE	95% CI	Coef.	SE	95% CI
Rural location	1.14	0.24	0.75, 1.72	1.09	0.16	0.81, 1.45	0.02	0.96	-1.88, 1.93	0.11*	0.05	0.01, 0.22
Bias	0.76	0.19	0.45, 1.27	0.59**	0.11	0.39, 0.87	− 2.23	1.32	− 4.84, 0.38	− 0.48***	0.07	− 0.62, − 0.33
Misinformation	0.56*	0.14	0.33, 0.94	0.54**	0.97	0.38, 0.77	− 3.57**	1.16	− 5.86, − 1.28	− 0.34***	0.06	− 0.46, − 0.21
Received training	0.82	0.11	0.62, 1.08	1.93***	0.18	1.60, 2.33	− 0.11	0.59	− 1.29, 1.05	0.15***	0.33	0.08, 0.22
Years in job	0.95***	0.13	0.92, 0.97	0.99	0.01	0.97, 1.02	0.12	0.06	− 0.00, 0.25	− 0.00	0.00	− 0.11, 0.00
Work hours	0.99	0.13	0.96, 1.01	0.99	0.01	0.97, 1.01	0.04	0.06	− .07, 0.16	− 0.00	0.00	− 0.01, 0.00
Female	0.47*	0.17	0.22, 0.98	0.80	0.21	0.47, 1.37	− 0.08	1.80	− 3.63, 3.45	− 0.04	0.10	− 0.24, 0.16
Age	1.00	0.01	0.97, 1.02	1.01	0.01	0.99, 1.03	0.07	0.07	− 0.06, 0.21	0.20	0.12	− 0.03, 0.43
Physician Assistant	0.99	0.43	0.41, 2.35	1.07	0.34	0.57, 2.01	3.62	2.12	− 0.55, 7.81	0.20	0.11	− 0.03, 0.43
Nurse Practitioner	0.95	0.40	0.41, 2.20	1.03	0.32	0.55, 1.92	7.55***	2.07	3.46, 11.64	0.30	0.12*	0.07, 0.53
Physician	Ref.	Ref.	Ref.	Ref.	Ref.	Ref.	Ref.	Ref.	Ref.	Ref.	Ref.	Ref.
White alone	0.73	0.34	0.29, 1.84	0.73	0.23	0.38, 1.39	− 0.87	2.10	− 5.01, 3.26	− 0.27	0.22*	− 5.02, 3.26

*Coef.* unstandardized regression coefficient; *OR* odds ratio, *SE* standard error; *95% CI* 95% confidence interval; coefficients are significant when the 95% CI does not include 0 and odds ratios are significant when the 95% CI does not contain 1.00. *p < 0.05, **p < 0.01, ***p < 0.001

## Discussion

Results of our state-wide survey indicated that a greater endorsement of buprenorphine misinformation was associated with lower willingness to work with patients with OUD, less interest in increasing the percentage of patients managed with OUD, a lower likelihood of having an X-waiver, and, among those without a waiver, less interest in receiving an X-waiver. Importantly, these findings held after controlling for relevant HCP demographics and known factors associated with treatment outcomes (e.g., training and explicit bias). Most important, buprenorphine misinformation was common, with nearly half of HCPs endorsing at least one piece of misinformation. These findings suggest that misinformation is an important barrier to overcome in terms of HCP interest in treating OUD and willingness to prescribe buprenorphine.

Although education of clinicians has decreased negative attitudes towards patients with OUD [32], the impact of education on treatment intentions or behaviors related to OUD has not received adequate empirical attention. Our findings are thus informative because we demonstrated that misinformation is not only prevalent among HCPs, but predicts integral outcomes related to OUD treatment, including willingness to treat OUD, an interest in taking on more patients with OUD, as well as using buprenorphine as measured by having enrolled in the X-waiver training course or having an intention to do so. These findings provide confidence that misinformation is important to address alongside other barriers to buprenorphine use, such as knowledge and bias.

Although misinformation regarding MOUD is likely related to stigma, interventions focused on decreasing negative attitudes towards people with OUD alone may not be enough to encourage buprenorphine use. Similarly, general knowledge improvement may help dispel myths that buprenorphine is ineffective but is unlikely to address concerns about buprenorphine being a partial opioid agonist, or that MOUD is not compatible with remission from OUD. Education is important; however, there are significant differences between being uninformed about the mechanism of MOUD and misinformed about its effectiveness or appropriateness for managing OUD. Future work is needed to understand the most common sources of buprenorphine misinformation, whether from colleagues, preceptors, or the general public, to better inform training intervention development.

Ultimately, our findings suggest that the best interventions to counteract misinformation will address underlying concerns and fears related to the medication. Although our study was conducted just prior to the removal of the X-waiver, these findings remain important. Decades of buprenorphine regulation [38] created considerable stigma around this medication. Evidence suggests that longstanding regulations reinforced the belief that this medication is complicated to prescribe, poses safety risks, and requires major changes in primary care practice to accommodate adjunctive counseling or urine drug testing [22, 31, 39, 40]. It is likely that these impacts will take time to overcome, and our study may serve as a baseline for understanding misinformation and its relationship to buprenorphine prescribing.

Because the X-waiver requirement had been weakened prior to our study and was removed altogether soon after the study was completed, it is possible that the outcome measuring interest in the X-waiver was affected by ongoing changes in regulation. Future studies should assess changes in buprenorphine prescribing after the removal of regulation, as well as changes in attitudes toward buprenorphine. A priority for future work is moving beyond identifying barriers to buprenorphine use, to develop new implementation strategies to increase buprenorphine prescribing, which can be tested using rigorous study designs.

Importantly, in developing new implementation strategies to increase buprenorphine use, we can learn from previous efforts to address misinformation among public health professionals. Effective tactics are needed to increase confidence with buprenorphine, particularly in the primary care setting, where it is vastly underutilized. Strategies to correct misinformation must come from reputable sources, as previous studies suggest that trusted individuals have the greatest impact on changing attitudes and behaviors [41]. Engaging professional organizations and scientific bodies to help dispel information is key, as is enlisting experts on addiction medicine or primary care to participate in training interventions. Although distrust in science and public health has grown among the public [42] , HCPs may not be as susceptible to these trends and may benefit from explicit efforts to correct misinformation from peers, experts, and professional organizations, alongside stigma-reduction and educational strategies.

## Limitations

Our study employed a large sample of HCPs currently working in primary care-aligned disciplines, to understand misinformation related to buprenorphine, yet important limitations exist. We did not use a probability-based sampling approach due to challenges faced with enrolling HCPs in the study. As such, our sample is not representative of all Ohio HCPs, and the findings may not be generalizable. We addressed this limitation by recruiting through multiple mechanisms to increase diversity in our sample. Further, although our survey was anonymous, responses about misinformation and bias are likely subject to social desirability bias, which could result in an underestimate of the prevalence of buprenorphine misinformation endorsed among HCPs. As a result, this limitation does not diminish confidence in our findings that misinformation is associated with OUD treatment attitudes or behaviors.

## Conclusion

Among HCPs currently practicing in primary care-aligned specialties in Ohio, misinformation is both prevalent and associated with lower willingness to treat OUD and prescribe buprenorphine, independent of previous training or stigma. Increasing HCP use of buprenorphine remains an important public health challenge, and our findings suggest that new training efforts are needed. Misinformation regarding buprenorphine is common among HCPs, and should be explicitly addressed in future implementation strategies, alongside ongoing efforts to provide education on buprenorphine prescribing guidelines and to reduce stigma. Understanding the concerns HCPs have with using buprenorphine, as well as misinformation they have received, is critical to increasing buprenorphine prescribing and access to this highly effective medication.

## Data Availability

Data are available from the study team upon reasonable request.

## Abbreviations

OUD: Opioid use disorder
PCP: Primary care professional
MOUD: Medication for opioid use disorder
PWID: Persons who inject drugs
HCP: Health care professional
